# Effects of Particle Size on the Shear Behavior of Coarse Grained Soils Reinforced with Geogrid

**DOI:** 10.3390/ma7020963

**Published:** 2014-02-07

**Authors:** Daehyeon Kim, Sungwoo Ha

**Affiliations:** Department of Civil Engineering, College of Engineering, Chosun University, 375 Seosuk-dong, Dong-gu, Gwangju 501-759, Korea; E-Mail: gilryong@nate.com

**Keywords:** direct shear test, geogrid, reinforcement effect, shear behavior, coarse grained soils

## Abstract

In order to design civil structures that are supported by soils, the shear strength parameters of soils are required. Due to the large particle size of coarse-grained soils, large direct shear tests should be performed. In this study, large direct shear tests on three types of coarse grained soils (4.5 mm, 7.9 mm, and 15.9 mm) were performed to evaluate the effects of particle size on the shear behavior of coarse grained soils with/without geogrid reinforcements. Based on the direct shear test results, it was found that, in the case of no-reinforcement, the larger the maximum particle size became, the larger the friction angle was. Compared with the no-reinforcement case, the cases reinforced with either soft geogrid or stiff geogrid have smaller friction angles. The cohesion of the soil reinforced with stiff geogrid was larger than that of the soil reinforced with soft geogrid. The difference in the shear strength occurs because the case with a stiff geogrid has more soil to geogrid contact area, leading to the reduction in interlocking between soil particles.

## 1. Introduction and Objective of the Study

In most cases, soils support the load of civil structure. In order to understand the characteristics of stress-strain and volume change of soils subjected to load, shear test should be performed. Through a shear test, we can understand how soils reach to the state of plastic failure as the load applied to them increases. The most important point for shear test is that we can obtain the shear strength parameters of soils necessary for the design of civil structures.

The engineering characteristics of soil vary depending on the size of its particle. Soils are categorized into clay (<0.002 mm), silt (0.075 mm–0.002 mm), sand (4.75 mm–0.075 mm), and gravel (76.2 mm–4.75 mm), based on the size of soil particle using the Unified Soil Classification System (USCS). Clay and silt are categorized into fine grained soil and sand and gravel are classified as coarse grained soil. In general, for the evaluation of shear behaviors of clay and sand, the small direct shear test is performed using the shear box with the size of 5 cm × 5 cm. In the case of gravel, which is larger than sand, we usually obtain shear strength parameters using the large direct shear test.

Coarse-grained soils, such as gravel, are used in many fields including earth dams, harbor facilities, atomic power waste storage areas, and roadbed materials. The particle sizes of coarse-grained soils used in the sites are in the range of some cm to some dozen cm. In many cases, the coarse grained soils which are larger than the gravel size of 76.2 mm, based on USCS, are used in the sites. In order to perform the shear test for the coarse grained soils with large particle sizes, we need to make a large shear box, as well as a large shear test device and, therefore, the cost of a shear test, in such case, will be quite significant. For this reason, research on the evaluation of shear behavior, using a large shear test, for coarse grained soils with large particles, is not yet fully satisfactory and further research is still needed.

In order to perform the direct shear test considering the particle size for coarse-grained soils with large particles, not only significant cost is required, but, also, many technical problems still exist. To solve the issues, shear characteristics of test materials have been identified so far mainly from the test results obtained through laboratory test for similar gradation samples of original gradation reduced down to similar gradation. These properties of coarse-grained soils obtained are applied to the design and analysis of actual ground structures. For the estimation of more accurate behavioral characteristics of ground structures, it is essential to identify the changes in shear behavior, depending on the particle size [1,2].

Recently, several studies on evaluating strength parameters by performing the direct shear test for coarse grained soils with relatively large particle size were done [3–5]. Even though large direct shear test devices are used for the direct shear test, the particle sizes of coarse grained soils, which are allowed for the devices, are generally very small compared to that used in actual sites, and, therefore, the test should be performed by reducing the particle size down to the level which the test device could allow.

It has been known that the characteristics of shear behavior of coarse-grained soils, with reduced particle size, are not as the same as those of coarse-grained soils with an actual size. Compared with other ground materials, coarse grained soils have significant differences as to the type of parent rock, particle size, the density of sample, and the crushing of particles pursuant to compaction energy. The influence of particle size on the shear strength on the size of particle diameter was known to have insignificant differences in the case of sand [6] whereas, in the case of coarse-grained soils with big particle diameters, there have been many different opinions arising among the researchers. There has been a deal of research carried out on the influence of particle size of coarse grained materials on shear strength [1–5,7–10], but consistent conclusions, as to the differences or influence, were not made among the researchers.

Lee *et al.* [5] evaluated the influence of maximum particle size and gradation distribution on shear behavior using coarse graining materials through the large direct shear test. Kim *et al.* [2] performed the triaxial compression test and direct shear test for decomposed granite soils and reported the results using gradation distribution curves, before and after the tests, that the maximum particle size and gradation distribution have effects on shear strength, shear strain, and volume change. Due to the limit in preparing test devices, depending on the particle size of coarse-grained soils, there are very few cases of studies regarding it.

The purpose of this research is to evaluate the influence of difference in maximum particle diameter on the shear behavior of coarse-grained soils reinforced with geogrid, based on large direct shear test for coarse-grained soils with different particle sizes.

### Method of Test Gradation

As mentioned earlier, the particle diameter of coarse-grained soils are in the range from some cm to some dozen cm, and, therefore, it is typical to reduce the particle diameter of a material to use for shear test (Maximum particle diameter of coarse grained soil should be below 1/8–1/10 of the size of test piece specimen). Gradation of coarse-grained soils is comprised of original gradation, test gradation and fill gradation. Among them, original gradation means actual material or gradation in the specification, test gradation means the gradation of material for shear tests, such as triaxial compression tests, and fill gradation means the gradation of the material actually used.

The following two methods are mainly used as methods for adjusting original gradation to test gradation:

- Parallel grading method;
- Scalping and replacement method.

#### (1) Parallel Grading Method

Parallel grading means the gradation obtained by parallel shift of gradation distribution curve as much as the limit of maximum particle diameter in line with the size of the testing device, and the gradation distribution curve is drawn pursuant to the definition of the formula (1):

$$D_{i}=D_{\mathrm{Bi}}/R$$ (1)

Here, D_i_: Particle diameter of similar gradation (mm);

D_Bi_: Particle diameter of original gradation (mm);

R: Similarity ratio.

The characteristics of parallel grading method is that its uniformity coefficient is the same as that of original gradation and its geometric arrangement is similar to that of original gradation. In this case the sample was prepared using similar gradation, however, the content ratio of granular powder shows a relatively increasing trend. In this research, test gradation was made using the parallel grading method in order to mix samples with similar C_u_ and C_c_ after determining three maximum particle diameters of coarse grained soils: 4.75 mm, 7.9 mm, and 15.9 mm.

#### (2) Particle Crushing of Coarse Grained Soils

When performing a direct shear test, coarse-grained soils may be crushed where particles in the coarse-grained soils are crushed by external load, such as compaction and shear. Crushing of particles gives a significant impact on the shear behavior of coarse-grained soils, and we need to evaluate the degree of crushing. In general, the larger the particle diameter, the more the load applied to the particle diameter, which results in the occurrence of the crushing of particles even under low confining pressure. Based on the results of a shear test for coarse grained soils, Yamamuro and Lade [11] reported that the degree of crushing of coarse-grained soils gives the most significant factor for shear strength at high level of confining pressure (*i.e.*, more than 2 MPa). In the case of coarse-grained soils with large particles, they are influenced by the shape, size, density, and compression strength of aggregate (Marsal [12]). In general, as soil particles have high resistance to compression forces, breaking, due to compression, does not occur unless a fairly significant compression load is applied. Accordingly, the shear strength of soil particles has the most significance when the shear strength occurs by friction force.

Depending on the degree of crushing of coarse-grained soils, the mechanical properties of coarse-grained soils may become different. The crushing nature of coarse-grained soils has a significant influence on compaction, and the crushing of particles at the time of compaction has the same effect as over-compaction and, thus, the strength of material will be rather reduced.

## 2. Laboratory Test Program

### 2.1. Contents of Direct Shear Test

In this research, the large direct shear test was performed for coarse-grained soils with maximum particle diameters of 4.75 mm, 7.9 mm, and 15.9 mm, using a large direct shear test device (Geotm, Seoul, Korea) (which was purchased by Chosun University for supporting the research) to identify shear behavior of coarse-grained soils for the cases of reinforcement with soft geogrid/stiff geogrid, as well as the case without reinforcement (Vertical load 96, 196, and 294 kPa). In order to enhance the reliability of this research, the tests for vertical load of 96 kPa were additionally carried out, 36 times in total.

### 2.2. Preparation of Sample

#### 2.2.1. Maximum Particle Diameter of Sample

The samples used in this research were taken from the Seomjin River. Maximum particle diameter of the samples taken was, maximum, 25 mm, but, for the tests, maximum particle diameters of samples prepared were 4.75 mm, 7.9 mm, and 15.9 mm considering the size of shear box (300 mm × 300 mm).

#### 2.2.2. Adjustment of Test Gradation

**Figure 1.**
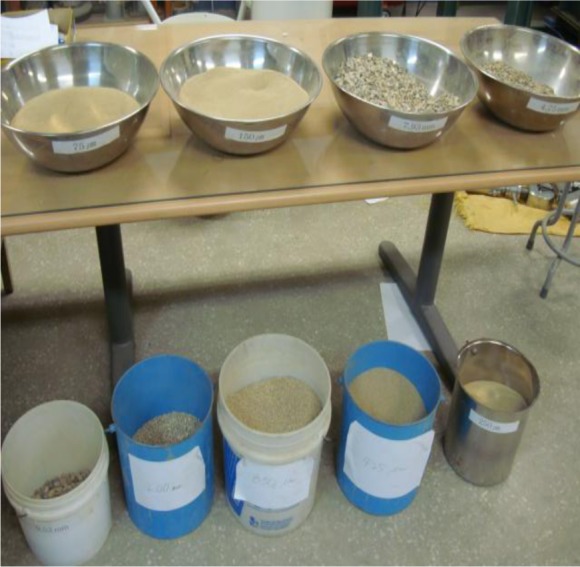
Classification of particle diameter through gradation test.

Gradation test for soil is performed for the purpose of classifying gradation distribution of materials, which is expressed as the ratio of weight percentage for respective particle diameters of coarse-grained soils, the test was performed in accordance with KS F 2302, and sieve analysis was performed using test piece specimens with 15.9 mm, 9.5 mm, 7.9 mm, 4.75 mm, 2.0 mm, 0.85 mm, 0.42 mm, 0.25 mm, 0.15 mm, and 0.075 mm to make the test gradation. Figure 1 is a picture for the classified soils by particle diameter through gradation test for the samples taken from the Seomjin River.

In order to see the effect of particle size, only on the internal friction angle (*i.e*., eliminating the influence of cohesion), the particle size of less than 0.075 mm was not used. A gradation distribution curve was drawn using coarse-grained soils with particle diameter of 4.75 mm–0.075 mm only, and, then, a test gradation distribution curve was shown by a diagram by moving the gradation distribution curve in parallel, using the parallel grading method for maximum particle diameters of 15.9 mm, 7.9 mm, and 4.5 mm. At this time, uniformity coefficient C_u_ and curvature coefficient C_c_ are shown in Table 1, and the sample, in which the maximum particle diameter of 4.75 mm of coarse-grained soils was included, is named as Sample A, while, naming Sample B, which included 7.9 mm in diameter, and Sample C, which included 15.9 mm in diameter.

**Figure 2.**
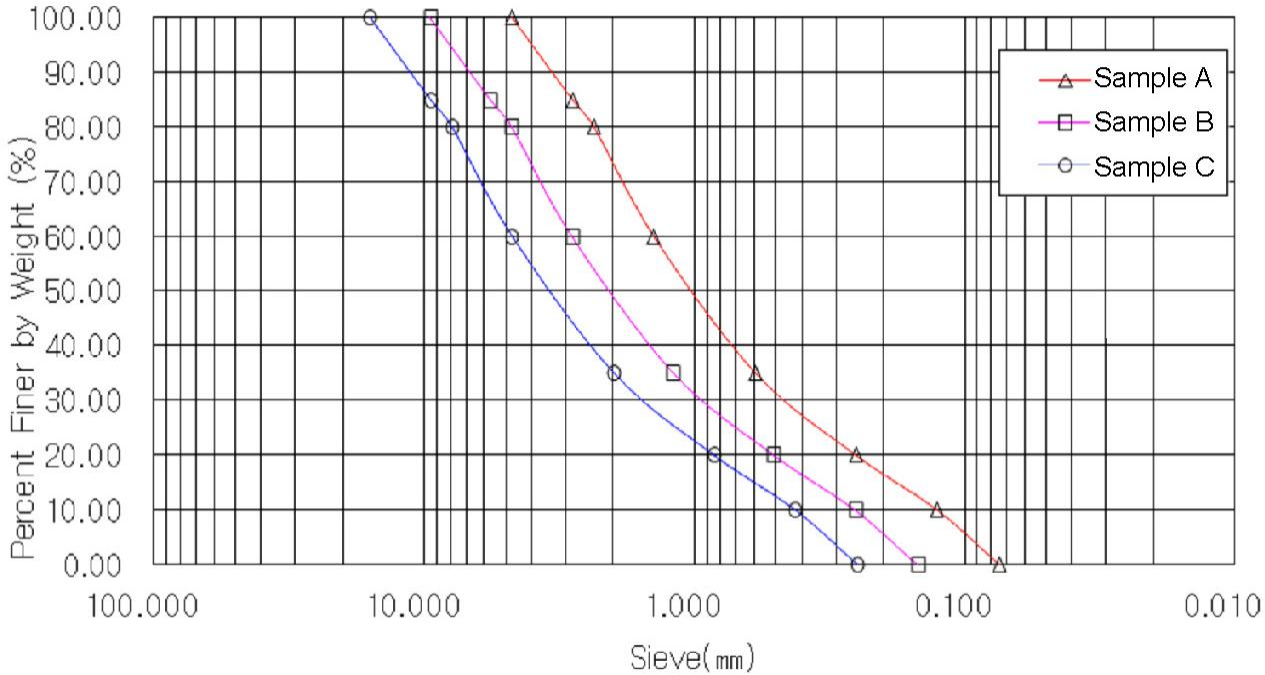
Gradation distribution curve applying parallel grading.

After drawing a gradation distribution curve for a maximum particle diameter of 7.9 mm (Sample B) of test samples at random, as shown in Figure 2, maximum particle diameter of 4.5 mm (Sample A) and that of 15.9 mm (Sample C) were adjusted, applying the parallel particle distribution method (Parallel grading). Table 1 indicates the properties of samples used. When classifying each sample, using USCS, the samples were classified as SW, which represents well-graded, and, uniformity coefficients, Cu, were 11.18 and 11.20 while curvature coefficients, C_c_, were 1.25 and 1.27. As the two coefficients were similar, basic and mechanical properties of respective particles will be similar.

#### 2.2.3. Loading of Vertical Load and Shear Load

After a loading pressure plate is carefully placed on the upper part of test specimen, targeted vertical loads are applied by test stage. In this research, three levels of vertical stresses of 98, 196, and 294 kPa, which were frequently used in the previous research, were applied to direct shear test for the purpose of comparison with previous research. Shear test was performed with a displacement speed of 1 mm/min at the time of loading shear load, and vertical load was controlled, to be maintained at a constant level, to avoid unnecessary vertical displacement in the course of shear test.

#### 2.2.4. Criteria for the Completion of Test

Shear test for sample was completed when horizontal displacement reached to 15%–20% to the length of shear direction of the test specimen. If peak shear stress is not shown until the time of additional displacement, for more than 5%, after reaching peak shear stress or peak shear stress is not clearly shown.

#### 2.2.5. Calculation of the Degree of Crushing

When a test was completed, shear load and vertical load are removed, the sample is moved to sample box, and then the gradation test was performed again for the analysis of the degree of crushing pursuant to shear test. The degree of crushing of particles was calculated by illustrating the results of tests in parallel with the original gradation distribution curve.

In this research, the degree of crushing was calculated using the difference in residual rates for representative particle diameter on the residual rate curve, before and after shear tests, in accordance with the definition of the degree of crushing proposed by Marsal (1967) [12], which is the most frequently used method for the calculation. That is to say, the crushing of a particle means the changing shape of gradation distribution as the sizes of particles are separated by the cause of breaking, and Marsal [12] proposed the following quantitative empirical formula (2) for the crushing of particles.

$$M=\frac{\sum \Delta f_{i}}{2}\times 100\%$$ (2)

Here, *M*: Degree of particle crushing; ∆*f_i_*: Difference in residual rate for representative particle diameter before and after crushing.

Here, the degree of particle crushing means the half of the total of differences of retained percentage for respective representative particle diameters, before and after crushing, on the retained percentage curve, which indicates the result of the crushing of gradation sample created for the initial test or the increase of granular powder due to the friction among particles and gradation redistribution.

### 2.3. Large Direct Shear Tester

**Figure 3.**
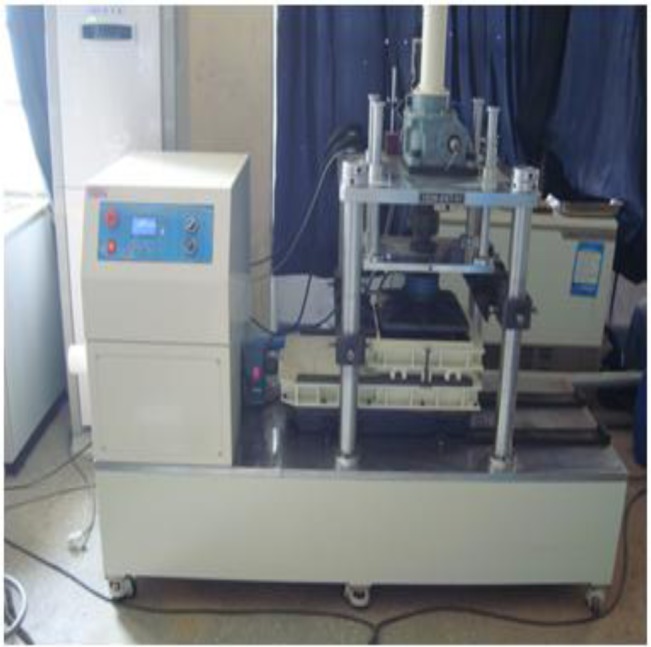
Large direct shear tester.

The large direct shear test device used for this research is a large scale test device (Geotm, Seoul, Korea), which has the capability of shearing of test specimens with the size of 300 mm × 300 mm, and the device can load vertical and horizontal axial direction loads up to maximum of 100 kN with a shear speed of 0.0001–10 mm/min using the SERVO MOTOR method. Figure 3 shows the large direct shear test device.

### 2.4. Test Method

**Figure 4.**
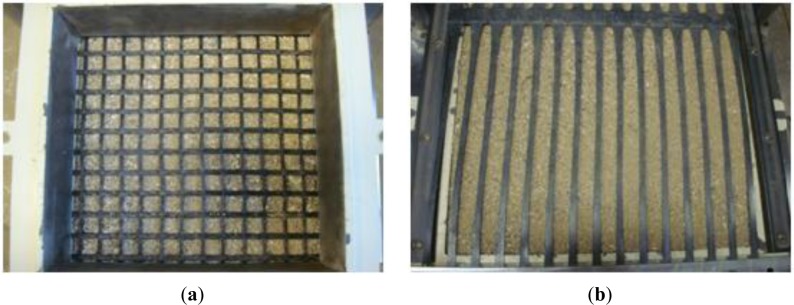
Geogrid was installed in the shear box: (**a**) Soft geogrid; (**b**) Stiff geogrid.

In this research, test samples were prepared by adjusting gradation to make maximum particle diameters of samples to 4.5 mm, 7.9 mm, and 15.9 mm applying parallel grading method. After compacting samples with a relative density of 70%, confining pressures were set at 98 kPa, 196 kPa, and 294 kPa, respectively, with a shear speed of 1 mm/min, and then the shear test was performed. At the time of compaction for the lower part of shear box, the compaction was done by dividing the box into two levels to ensure uniform gradations in the upper level, as well as lower level of the box. For the last compaction layer, the upper side was leveled so that the pressure plate is not inclined. For the shear test for coarse-grained soils, reinforced with a geogrid, compaction for lower part was done first, a geogrid was installed on it, and then compaction for the upper part of shear box was done. Figure 4 shows the installation of a geogrid in the shear box. The following procedures were done to fix the geogrid. First, we put aggregates in the lower shear box and placed the geogrid. Then, we fixed the end of the geogrid using a steel bar. After that, we placed the upper shear box and filled the aggregates.

Shear test for sample was completed when horizontal displacement volume reached 15%–20% to the length of shear direction of the test piece specimen if peak shear stress is not shown until the time of additional displacement for more than 5% after reaching to peak shear stress or peak shear stress is not clearly shown.

## 3. Results of Large Direct Shear Tests and Discussions

### 3.1. Result of Shear Test

“Shear strain” is defined as a percentage of the quotient of shear strain divided by the length of shear box. When maximum value of shear stress was generated from the relationship of shear stress-shear strain, it was used as the maximum shear stress value, and in case the maximum value was not generated, the value at the time when horizontal shear strain is 15% was selected as the maximum shear stress value.

#### 3.1.1. Sample with Maximum Particle Diameter of 4.5 mm

**Figure 5.**
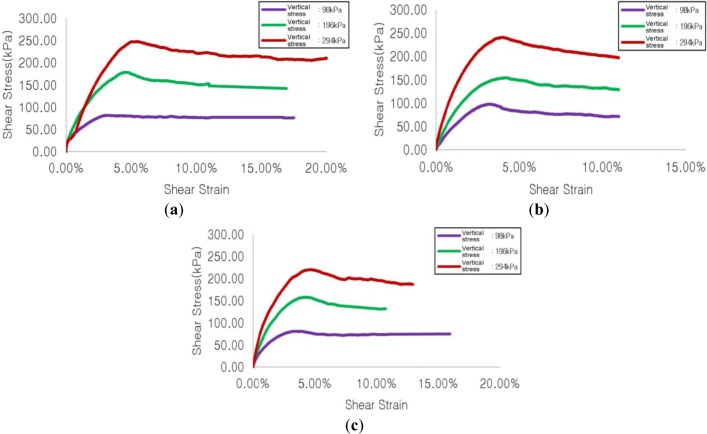
Results of shear test for maximum particle diameter of 4.75 mm. (**a**) No reinforcement; (**b**) Reinforcement with soft geogrid; (**c**) Reinforcement with stiff geogrid.

Figure 5a–c show the shear stress-shear strain for the cases of no reinforcement, reinforcement with soft geogrid, and reinforcement with stiff geogrid, respectively. As we can see from Figure 5a–c, shear stress has a tendency of showing constant value after reaching its maximum value, and it is the typical shear behavior of general coarse grained soils. At the level of vertical stress of 98 kPa, we found that the value becomes flat after reaching the maximum shear stress, whereas, at the confining pressure levels of 196 kPa and 294 kPa, the value was converging with a steeper slope than 98 kPa after reaching the maximum value. The shear stress, when reinforced with soft geogrid, was increased by 4% at the time of vertical stress of 98 kPa, compared to that in the case of no reinforcement, while showing decrease by 11%, respectively, at vertical stresses of 196 kPa and 294 kPa. The shear stress, when reinforced with stiff geogrid, was increased by 26% at a vertical stress of 98 kPa, compared to that in the case of no reinforcement while showing decreases of 14% and 3%, respectively, at vertical stresses of 196 kPa and 294 kPa.

#### 3.1.2. Sample with Maximum Particle Diameter of 7.9 mm

**Figure 6.**
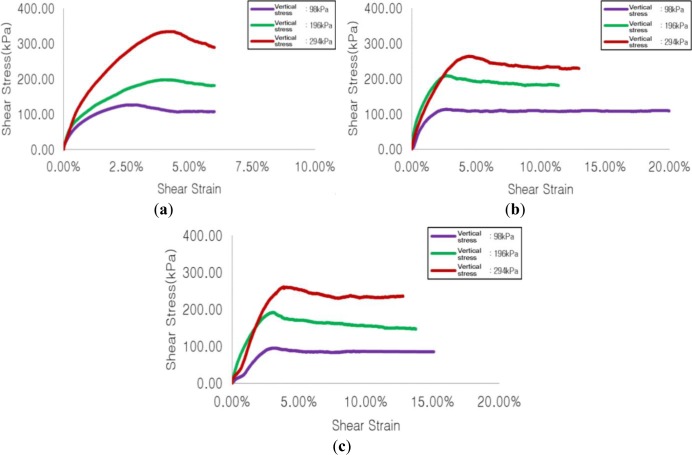
Results of shear test for maximum particle diameter of 7.9 mm. (**a**) No reinforcement; (**b**) No reinforcement; (**c**) Reinforcement with stiff geogrid.

Figure 6a–c show shear stress-shear strain for the cases of no reinforcement, reinforcement with soft geogrid, and reinforcement with stiff geogrid, respectively. The maximum shear stress generated in the case of reinforcement with soft geogrid was increased by 6% at a vertical stress of 196 kPa compared to that in the case of no reinforcement, while showing decreases of 15% and 21%, respectively, at the time of vertical stresses of 98 kPa and 294 kPa. The maximum shear stress generated when reinforced with stiff geogrid was decreased by 25%, 3%, and 22%, respectively, at vertical stresses of 98 kPa, 196 kPa, and 294 kPa, compared to that in the case of no reinforcement.

#### 3.1.3. Sample with Maximum Particle Diameter of 15.9 mm

**Figure 7.**
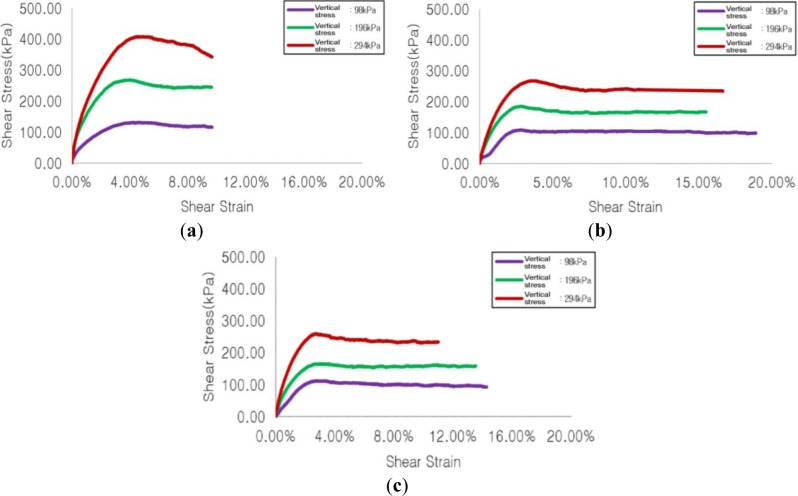
Results of shear test for maximum particle diameter of 15.9 mm. (**a**) No reinforcement; (**b**) Reinforcement with soft geogrid; (**c**) Reinforcement with stiff geogrid.

Figure 7a–c respectively show shear stress-shear strain for the cases of no reinforcement, reinforcement with soft geogrid, and reinforcement with stiff geogrid. Maximum shear stress generated in the case of reinforcement with soft geogrid was decreased by 22%, 31%, and 34%, respectively, at vertical stresses of 98 kPa, 196 kPa, and 294 kPa, compared to that in the case of no reinforcement, and maximum shear stress generated when reinforced with stiff geogrid was decreased by 15%, 38%, and 37%, respectively, at vertical stresses of 98 kPa, 196 kPa, and 294 kPa.

### 3.2. Shear Strength Parameters

Table 2 indicates shear strength parameters, such as interface friction angle and adhesion, for no reinforcement, reinforcement with soft geogrid, and reinforcement with stiff geogrid. In the case of no reinforcement, as the maximum particle diameter became larger, the more the internal friction angle was increased, but the internal friction angle in the case of reinforcement with geogrid turned out to be smaller than that in the case of no reinforcement. The tendency of decrease in the interface friction angle due to reinforcement with geogrid is similar to the results of tests in the previous research done by Seo *et al.* [4].

The values presented in Table 2 defined that the interface friction angle at the contact surface of soil-geogrid, divided by internal friction angle of soil-soil, is friction coefficient. When reinforcement was done using soft and stiff geogrid for the sample with maximum particle diameter of 4.5 mm, shear strengths of 97% and 93% were revealed, shear strengths of 87% and 83% were revealed in the case of sample with maximum particle diameter of 7.9 mm, and 68% and 64% in the case of 15.9 mm. In the case of reinforcements with soft and stiff geogrid, friction efficiencies were decreased in both cases as maximum particle diameters were increased. It appears that the maximum shear stress became smaller due to the decrease in interlocking in between particles as maximum particle diameters were increased. A comparison of both, reinforcements with soft geogrid, and stiff geogrid, showed that the friction efficiency of stiff geogrid was decreased more compared to that of soft geogrid. From this result, we found that, in the case of no reinforcement, friction in between particles increased whereas there are more sliding phenomena occurred as the stiffness of geogrid was increased, which resulted in a decrease in maximum shear stress. As all other conditions for the tests were the same, except for the size of particle diameters, such differences in the shear behavior of coarse-grained soils reinforced with geogrid were generated due to the differences in the size of the particles.

In the case of reinforcement with soft/stiff geogrid, the more the maximum particle diameter was increased, the more adhesion force was increased. In the case of reinforcement with soft geogrid, adhesion of sample B was higher than that of sample A by 31.47%, while that of sample C was higher than that of sample A by 47.18%. In the case of reinforcement with stiff geogrid, adhesion of sample B was higher than that of sample A by 64.99%, while that of sample C was larger than that of sample A by 79.42%. Such differences were generated because the sliding of soil particles from the surface of geogrid was increased due to the increase in the contact area ratio of geogrid.

### 3.3. Degree of Crushing

#### 3.3.1. Sample with Maximum Particle Diameter of 4.5 mm

**Figure 8.**
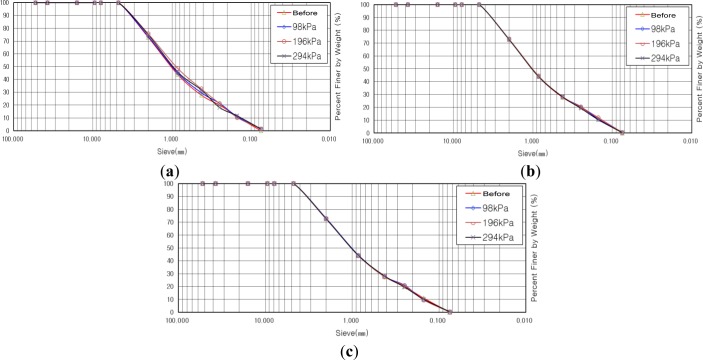
Gradation distribution curve before and after shear test with maximum particle diameter of 4.5 mm. (**a**) No reinforcement; (**b**) Reinforcement with soft geogrid; (**c**) Reinforcement with stiff geogrid.

Figure 8a–c indicate gradation distribution curves before and after shear tests for the sample with maximum particle diameter of 4.5 mm. The result of the calculation for the degree of crushing revealed that the degree of crushing was increased as vertical stress was increased. We estimated that the degree of crushing would be decreased in the case of reinforcement with soft/stiff geogrid at a vertical stress of 98 kPa, but it was increased by 0.35% compared to that for the case of no reinforcement. The reason for such a small increase, of 0.35%, was due to the differences in relative density from the result of different compaction of the shear box in the course of preparation for the shear test. In the case of reinforcement with soft geogrid at vertical stresses of 196 kPa and 294 kPa, the degrees of crushing were decreased by 0.1% and 0.9%, respectively, while showing decreases of 0.1% and 0.7%, respectively, in the case of reinforcement with stiff geogrid with the same conditions for vertical stress. All the results show an insignificant decrease in the degree of crushing, of less than 1%, but we can say that the result shows the effect of the geogrid as, at least, the degree of crushing is decreased by using geogrid.

#### 3.3.2. Sample with Maximum Particle Diameter of 7.9 mm

**Figure 9.**
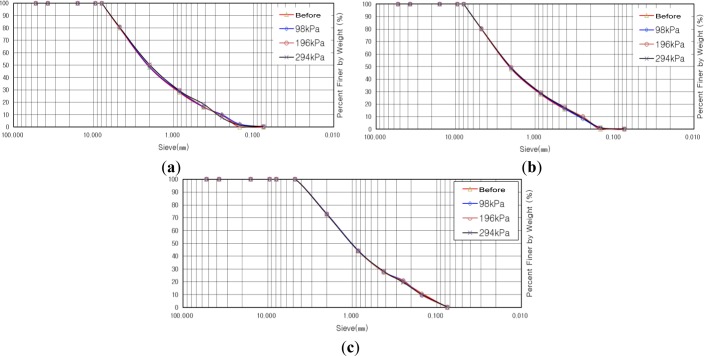
Gradation distribution curve before and after shear test with maximum particle diameter of 7.9 mm. (**a**) No reinforcement; (**b**) Reinforcement with soft geogrid; (**c**) Reinforcement with stiff geogrid.

Figure 9a–c indicate gradation distribution curves, before and after shear tests, for the samples with a maximum particle diameter of 7.9 mm. The result of the calculation for the degree of crushing revealed that the degree of crushing was increased as vertical stress was increased, similar to the case of a maximum particle diameter of 4.5 mm. The degree of crushing, in the case of reinforcement with soft geogrid at a vertical stress of 98 kPa, was increased by 0.2% compared to that of the case of no reinforcement, while showing an increase of 0.6% in the case of reinforcement with stiff geogrid. In the case of reinforcement with soft geogrid, at vertical stresses of 196 kPa and 294 kPa, the degrees of crushing were decreased by 0.5% and 0.2%, respectively, while by 0.9% and 0.7%, respectively, in the case of reinforcement with stiff geogrid with the same conditions for vertical stress. From the results, we found that the tendency for crushing for the sample with a maximum particle diameter of 7.9 mm is similar to that for a maximum particle diameter of 4.5 mm.

#### 3.3.3. Sample with Maximum Particle Diameter of 15.9 mm

**Figure 10.**
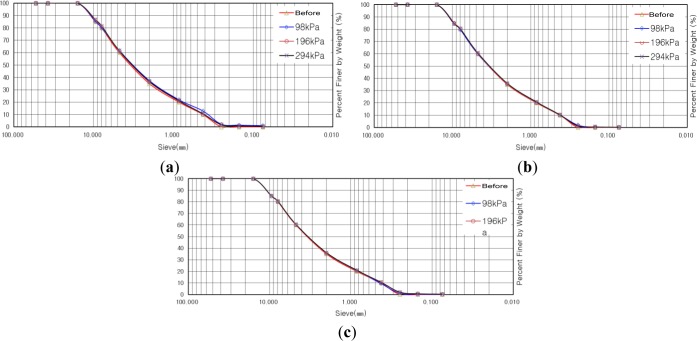
Gradation distribution curve before and after shear test with maximum particle diameter of 15.9 mm. (**a**) No reinforcement; (**b**) Reinforcement with soft geogrid; (**c**) Reinforcement with stiff geogrid.

Figure 10a–c indicate the gradation distribution curves, before and after shear tests, for the sample with a maximum particle diameter of 15.9 mm. The result of the calculation for the degree of crushing revealed that the degree of crushing was increased as vertical stress was increased, similar to the case of a maximum particle diameter of 4.5 mm and 7.9 mm. In the case of reinforcement with soft and stiff geogrid, differently from the tendency in the cases of maximum particle diameters of 4.5 mm and 7.9 mm, the degrees of crushing, in the case of a maximum particle diameter of 15.9 mm, at all vertical stresses of 98 kPa, 196 kPa, and 294 kPa, were all decreased within the range of 0.5%–3.2% compared to that for the case of no reinforcement with the same vertical stresses. From the results, we found that the tendency for crushing for the sample with a maximum particle diameter of 7.9 mm is similar to that in the cases of maximum particle diameters of 4.5 mm and 7.9 mm and the decrease in the degree of crushing was larger in the case of a maximum particle diameter of 15.9 mm.

#### 3.3.4. Influence of Maximum Particle Diameter and the Types of Geogrid on the Degree of Crushing

Table 3 indicates the influence of maximum particle diameter on the degree of crushing for no reinforcement, reinforcement with soft geogrid, and reinforcement with stiff geogrid, respectively.

We can see that, in the case of no reinforcement, the degree of crushing is increased as the size of particle is increased. For each sample, the degrees of crushing for both cases, using soft geogrid and stiff geogrid, did not show significant differences, but the case of stiff geogrid generated a minor increase in the degree of crushing compared to the case of soft geogrid. It was confirmed that geogrid could prevent crushing for the cases of no reinforcement and reinforcement with geogrid.

#### 3.4. Influence of Degree of Crushing on Vertical Stress and Shear Stress

**Figure 11.**
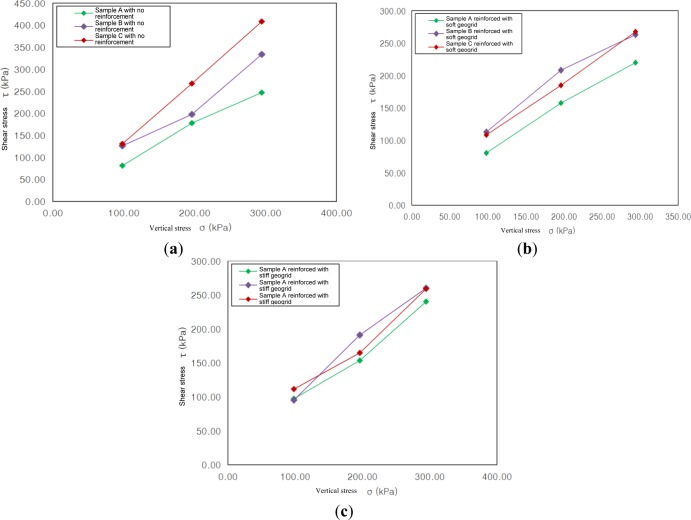
Relationship of shear stress—vertical stress. (**a**) The case of no reinforcement; (**b**) The case of reinforcement with soft geogrid; (**c**) The case of reinforcement with stiff geogrid.

Figure 11 indicates the relationship between vertical stress and shear stress, depending on maximum particle diameters for the cases of no reinforcement and reinforcement with soft/stiff geogrid.

In the case of no reinforcement, maximum shear stress for sample B, with a maximum particle diameter of 7.9 mm was increased by 10.83%–70.04% compared to that of sample A, with a maximum particle diameter of 4.75 mm, while the maximum shear stress for sample C, with a maximum particle diameter of 15.9 mm, was increased by 50.49%–70.04% compared to that of sample A. For the case of reinforcement with soft geogrid, the maximum shear stress of sample B was increased by 19.70%–33.88% compared to that of sample A, while sample C was increased by 17.22%–27.13% compared to that of sample A. In the case of reinforcement with stiff geogrid, sample B was increased by 2.17%–24.52% compared to sample A, while sample C was increased by 7.29%–14.91% compared to sample A. When vertical stresses were 196 kPa and 294 kPa, the maximum shear stress of sample C was smaller than that of sample B by 4.10% and 11.23%, respectively, in the case of reinforcement with soft geogrid, while sample C was smaller than sample B by 13.84% and 0.47%, respectively, in the case of reinforcement with stiff geogrid.

In conclusion, as all other conditions, except for the size of the particle diameters, were the same when performing the shear test, such differences in shear behavior appears to be due to the difference in the sizes of the particles.

## 4. Summary and Conclusions

In order to analyze the influence of the particle diameter of a sample on the shear strength of coarse-grained soils, large direct shear tests were performed on the coarse-grained soils with maximum particle diameters of 4.5 mm, 7.9 mm, and 15.9 mm for the cases of no reinforcement and reinforcement with soft/stiff geogrid by applying the parallel grading method. Based on the results of the shear tests, the following conclusions could be drawn:

(1) In the case of no reinforcement, the more the maximum particle diameter was increased, the more the maximum shear stress was increased. The same tendency was observed in the case of reinforcement with both soft geogrid and stiff geogrid. This indicates that the larger the particle size is, the higher the shear strength is.
(2) Interface friction angles of reinforcement, with soft and stiff geogrid, were smaller than that of no reinforcement. Such a difference was due to the increase in the area ratio where soil and geogrid are in contact, and the increase in the reduction of interlocking between soil particles.
(3) Friction efficiency was reduced as the maximum particle diameter was increased. As all other conditions were the same, except for the size of the particle diameter, the differences in shear behavior of coarse-grained soils, reinforced with geogrid, were due to the differences in the size of the particles.
(4) In the case of reinforcement with geogrid (soft/stiff geogrid), adhesion was increased as maximum particle diameter was larger. Adhesion generated, in the case of reinforcement with stiff geogrid, was larger than that generated in the case of reinforcement with soft geogrid. Such differences in adhesion force are due to an increase in the sliding of soil particles on the surface of geogrid, as the area ratio where the contact area of soil particle and geogrid surface is increased.

## Figures and Tables

**Table 1.** Physical properties of test samples.

Properties	Sample A	Sample B	Sample C
Maximum particle diameter (mm)	4.75	15.9	7.9
G_S_	2.610	2.622	2.638
e_max_	0.821	0.761	0.754
e_min_	0.419	0.380	0.403
Cu	11.17	11.20	11.18
C_c_	1.28	1.25	1.27
Unified Soil Classification System	SW	SW	SW

**Table 2.** Internal friction angle and cohesion.

Sample	Condition	Interface friction angle (°)	adhesion(kPa)
Sample with maximum particle diameter of 4.75 mm	No reinforcement	40.56	0
	Reinforcement with soft geogrid	39.34	13.12
	Reinforcement with stiff geogrid	37.57	20.85

Sample with maximum particle diameter of 7.9 mm	No reinforcement	47.97	0
	Reinforcement with soft geogrid	43.75(0.87)	17.25
	Reinforcement with stiff geogrid	42.51(0.83)	34.40

Sample with maximum particle diameter of 15.9 mm	No reinforcement	54.04	0
	Reinforcement with soft geogrid	42.90(0.68)	19.31
	Reinforcement with stiff geogrid	41.59(0.64)	37.41

**Table 3.** Influence of maximum particle diameter on the degree of crushing.

**Degree of Crushing** (%)	**Condition**		**Vertical Stress**		
			**98 kPa**	**196 kPa**	**294 kPa**

	**A**	No reinforcement	0.65	1.4	2.2
		Reinforcement with soft geogrid	1.0	1.3	1.3
		Reinforcement with stiff geogrid	1.0	1.4	1.5

	**B**	No reinforcement	0.8	2.0	2.5
		Reinforcement with soft geogrid	1.0	1.5	1.6
		Reinforcement with stiff geogrid	2.5	1.8	1.8

	**C**	No reinforcement	2.2	4.0	5.0
		Reinforcement with soft geogrid	1.7	1.9	1.8
		Reinforcement with stiff geogrid	1.7	1.8	2.1
